# Conclusion of the Report of the Proceedings of the Forty-Fourth Annual Meeting of the Michigan State Dental Association

**Published:** 1900-09-15

**Authors:** 


### CONCLUSION OF THE REPORT OF THE PROCEEDINGS OF THE FORTY-FOURTH ANNUAL MEETING OF THE MICHIGAN STATE DENTAL ASSOCIATION.

(*Continued from August No., page* 429.)

DR. FOWLER: I congratulate myself on being so gently treated, I expected to be flopped over and fired upon on both sides. The first thing I want to do is to square myself with the preacher. It was a preacher who married me, and I want to be the last fellow who has anything to say against him. There is no one for whom I have greater admiration than a preacher.

I am disappointed somewhat in the discussion. The point which I thought to be the strongest in the paper was that of manual training. Next to the literary it is the most important, and I am disappointed that it was not touched upon.

I do not know that I have anything further to say, but I had hopes that the point of manual training in the public schools would be brought out.

DR. DIACK: Just a few suggestions regarding the discussion which Dr. Hoff and Dr. Root brought out. Regarding the entrance requirements, either for examination by State Board or for entrance into our colleges. This is a hobby of mine, I may be mistaken, but the chances are I am not. I think our State University ought to stand for the highest; it is going to advance by following the example of the best schools in our country which are turning out the best men.

It should be the aim of public institutions to insist on certain requirements. And I should like to see the privilege of taking an examination for entering done away with and make it compulsory for the applicant to have a diploma from a high school. And looking forward into the future of dentistry, I think this will come.

I may be off on this, but that is one thing I insist upon. I do think our public institutions should hold the highest position. I think we ought to insist that they take that position.

So far as the requirements for taking examinations from the State Board, I am democratic enough to think that any man who is capable of passing the State Board or who shows sufficient skill in things required, can not be kept from taking the examination by any State law. I do not believe such a law constitutional, or could be carried out.

